# Architects' Description of the Buildings

**Published:** 1914-08-01

**Authors:** 


### A Description of the Buildings.

By THE ARCHITECTS TO BRISTOL INFIRMARY.

The position, the only available one, could scarcely be bettered. It affords uninterrupted sunshine and air and an excellent outlook. The plan is governed by the requirements of the most important parts of the new building—viz., the wards, which are placed axially north and south so as to receive sun all day.

Extensive accommodation for the various departments has been provided on the floors below the wards.

On the basement floor—and by "basement" it must not be understood that this floor is below the ground—will be found the following :—

The dental department, containing an entrance from Commercial Road; porter's room; large waiting-rooms for men and women respectively; two surgeries; recovery room; conservation room, arranged for eight chairs, in the first instance, with space for increasing the number to twelve, or more, when required; also a work-room and offices. On this floor also are the dining-room accommodation for sisters and nurses, and also for laundrymaids, and the new laundry, which consists of receiving rooms, washhouse, drying department, with the ordinary modern dry-closet system, through which hot air is blown by an electrically-driven fan, also a place for the slower drying of woollens; finishing room; airing room; and room for sorting and packing. The laundry appliances are by Messrs. T. Bradford and Co.

### Ground and Upper Floors.

On the ground floor are six suites of private sitting-rooms and bedrooms for resident officers; officers' smoking and common rooms; mess rooms for men students and lady students, and also for the matron and assistant matron; private room for the visiting staff; registrar's room; and sundry offices.

The first floor is devoted to women's medical 25-bed unit, comprising a ward of twenty-two beds, with sun balcony at the south end; one single-bed ward and one two-bed ward; sister's room; ward kitchen; larder; and the necessary rooms for storing linen and patients' clothes; also a sanitary wing, containing the usual equipment. On the same floor there is also a clinical laboratory.

The second floor is potentially another 25-bed unit, as it is capable of being converted so as to provide accommodation practically identical with that on the first floor, but it is now arranged as a maternity ward for twelve beds—ten in a large ward and two in a separate ward. It contains a day room, which room can serve a double purpose, it being possible to wheel the babies' cots into it at night. At the south end is an open-air balcony large enough to contain all the beds if desired.

The administrative portion of the ward is much the same as on the floor below, but contains a room for washing the babies; an operating-room; a room where patients may change and bathe upon arrival, and also a changing-room for the surgical staff.

On the roof of the ward is a garden, or airing ground, for the women patients. A fire-escape staircase is provided at the southern end of the ward, communicating from the roof-garden and from each floor-level to the ground.

At third-floor level the nurses' sleeping accommodation has been extended by the provision of twenty-two additional rooms, some of which have been obtained by the extension of the top storey of the old building, in which changes tending to improvement have been made.

### Construction.

The building is constructed of ferro-concrete upon the Hennebique principle. It stands on concrete pillars carried down to the rock, which lies at a depth of about thirty feet below the ground, and the construction is arranged so that, for the most part, it would be possible to clear away all the internal partitions and build others in new positions without in any way interfering with the main construction of the building. The floors of the wards are of teak boards.

For the wall surfaces glazed material has been used somewhat sparingly. In most cases the surface is of Keene's cement, which it is intended to finish with enamel. In order to reduce to a minimum the cleaning of brass-work, where possible taps are of metallic enamel.

The heating, ventilating, and hot-water service installations and water-softening plant have been carried out by Messrs. G. N. Haden and Sons, of Trowbridge. The heating is by low-pressure hot water, the circulation being accelerated by means of electrically-driven pumps. In the large wards the warming is supplemented by open fires in the centre of the floor.

The radiators are of the "Hospital" type, with fresh-air inlets at the back, fitted with internal shutters for regulating the admission of fresh air, and designed so as to be readily removable for cleansing.

The ventilation is by natural means, including the opening of windows. Extraction shafts are carried up the sides of the building. The interior of these is readily accessible, and can be hosed down. The operating-theatre alone is ventilated by mechanical means—viz., an electric fan, to effect an interchange six times in an hour.

With regard to the service of hot water, a considerable amount of work has been done apart from that involved by the erection of the new building. The hot-water service plant of the whole hospital has been centralised, and has been converted into what is known as an indirect system. This work has been done with a view to effecting economy in annual expenditure, and at the same time to obtain higher and more uniform temperatures. The primary water is heated by being passed through steam calorifiers arranged so as to utilise the heat from the exhaust steam of the laundry engine and pumps, live steam being introduced to make up the deficiency when required.

A new economiser of the "Green" type has been arranged in connection with the steam boiler, so that the maximum amount of heat may be extracted from the flue gases. Both the primary and secondary circuits are provided with accelerators for ensuring an ample flow of water. Tests recently made have shown an economical record which more than justifies the capital outlay incurred.

A water-softening plant has been provided, having a capacity of 1,000 gallons per hour. The softened water is used for the hot-water supply, laundry purposes, and boiler feed make-up. It is estimated that the introduction of this plant will save something like 50 per cent. in the cost of soap and 35 per cent. in the cost of soda.

The building could scarcely be plainer or more simple than it is, there being no architectural embellishments. The turrets along the sides are chimneys grouped together around the ventilation shafts. The flues can be swept from outside without bringing the soot through the rooms.

A new pathological department has been erected on land behind the Museum. The architects are Messrs. Oatley and Lawrence.

